# Complexes With Biologically Active Ligands. Part 2. Preparation of Copper(II) Complexes of Positively-Charged Derivatives of Aminoglutethimide

**DOI:** 10.1155/MBD.1996.57

**Published:** 1996

**Authors:** Claudiu T. Supuran

**Affiliations:** 1 University of Florence Laboratory of Inorganic and Bioinorganic Chemistry Via Gino Capponi 7 Firenze I-50121 Italy

## Abstract

Cu(II) complexes of 1-[4-(3-ethyl-piperidine-2,6-dione)-3-yl]-phenyl-2,4,6-trisubstituted pyridinium perchlorates, containing alkyl, aryl and combinations of these two types of moieties in their molecule were synthesized and characterized by elemental analysis, spectroscopy, magnetic, thermogravimetric and conductimetric measurements. In these complexes, Cu(II) ions are in octahedral geometry with four water molecules occupying the equatorial coordination sites and the two organic ligands in deprotonated state the remaining axial ones. The donor atom of these ligands is constituted by the ionized nitrogen of the glutarimide moiety. The new derivatives possess weak inhibitory activity towards the zinc enzyme carbonic anhydrase.


### COMPLEXES WITH BIOLOGICALLY ACTIVE LIGANDS. Part 2. PREPARATION OF COPPER(II) COMPLEXES OF POSITIVELY-CHARGED DERIVATIVES OF AMINOGLUTETHIMIDE

Claudiu T. Supuran
University of Florence, Laboratory of Inorganic and Bioinorganic Chemistry,
Via Gino Capponi 7, I-50121, Firenze, Italy

**Abstract**: Cu(II) complexes of 1-[4-(3-ethyl-piperidine-2,6-dione)-3-yl]-phenyl-2,4,6-trisubstituted pyridinium perchlorates, containing alkyl, aryl and combinations of these two types of moieties in their molecule were synthesized and characterized by elemental analysis, spectroscopy, magnetic, thermogravimetric and conductimetric measurements. In these complexes, Cu(II) ions are in octahedral geometry with four water molecules occupying the equatorial coordination sites and the two organic ligands in deprotonated state the remaining axial ones. The donor atom of these ligands is constituted by the ionized nitrogen of the glutarimide moiety. The new derivatives possess weak inhibitory activity towards the zinc enzyme carbonic anhydrase.

### Introduction

A large series of biologically active coordination compounds containing heterocyclic sulfonamides as ligands were reported recently [2-4]. Such derivatives behave as potent inhibitors of the ubiquitously spread zinc enzyme carbonic anhydrase (CA, EC 4.2.1.1) [2a,5] and since ligands such as acetazolamide **1**, methazolamide **2** or ethoxzolamide **3** are widely used pharmacological agents in the treatment of a variety of disorders, [5,6] presumably, their complexes might lead to the development of novel types of such drugs.[2a] In addition to these classical CA inhibitors, complexes were also prepared from positively-charged sulfonamides of type **4** (developed as isozyme-specific inhibitors for membrane-bound CA isozymes [7]) as

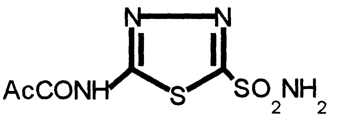

**1**

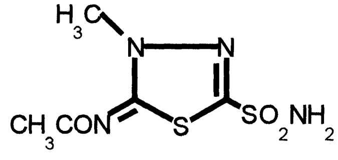

**2**

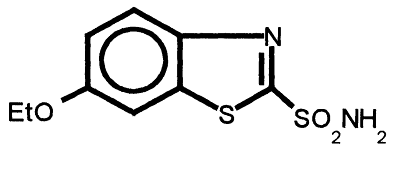

**3**

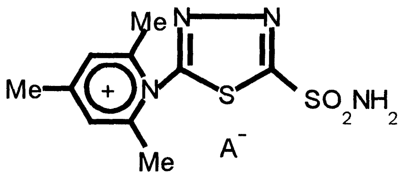

**4**

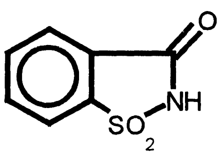

**5**

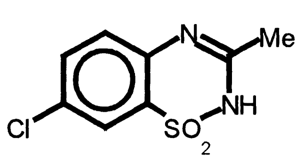

**6**

well as saccharin **5** (for which CA inhibitory properties were recently reported[8]) or diazoxide **6**, a compound possessing a completely different type of biological activity, but a coordination chemistry similar to that of compounds **1-4**. [1]

All these ligands coordinate transition- or main-group metal ions by means of the sulfonamido nitrogen atoms, in a monodentate or bidentate fashion (in which case endocyclic atoms such as the thiadiazolic N-3 or N-4 of acetazolamide **1**, or the nitrogen from the benzothiazole ring of ethoxzolamide **3**, participate by interaction with the cations too). [2-4] One of their specific features is the presence of a rather acidic NH proton in their molecule, with $pK_a$'s in the range 7.0 - 9.5 in derivatives **1-4** and **6** (saccharin **5** being a much stronger acid, with $pK_a$ of 1.3).[9] As a consequence, ligands of type **1-6** generally coordinate metal ions in their deprotonated state. [2-4] In addition to studies previously mentioned by us,[2-4] few other metal complexes were reported containing this type of ligands. A notable exception is constituted by the Cu(II) complexes of arylsulfonylated amino acids, such as tosylglycine[10] or the 4-aminophenylsulfonyl derivatives of glycine, alanine, valine and glutamic acid. [11]

The interesting biological activity of complexes containing such ligands, [2-4,10,11] as well as the scarcity of literature data in this field, prompted us to extend the studies to related systems, containing a rather acidic NH group, because of the presence of $SO_2$ or CO moieties in its vicinity.

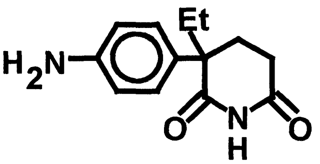

**7**

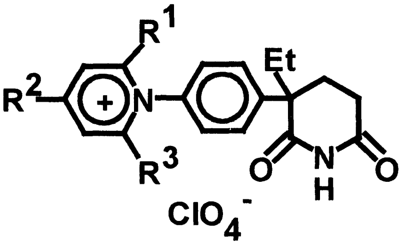

**8**

Aminoglutethimide (3-(4-aminophenyl)-3-ethylpiperidine-2,6-dione, **7**) derivatives, such as the positively-charged compounds of type **8**, recently reported,[12] appeared as interesting candidates for several reasons. Thus, aminoglutethimide is a clinically used drug in the treatment of breast and prostate cancer,[13] adrenocortical carcinoma [14] and Cushing syndrome [15] among others. It inhibits the conversion of cholesterol to 20-α-hydroxycholesterol, inhibiting in fact the cytochrome P-450 hydroxylation of this substrate. [16] In this way the first step of steroidogenesis is inhibited, a fact that explains the efficiency of the drug in the treatment of tumors sensitive to such hormones.[13-16] On the other hand, the NH proton in derivatives of type **8** is sufficiently acidic ($pK_a$-s in the range 9.0-9.6 [12]) to allow easy deprotonation and participation in interactions with metal ions, similarly with derivatives of types **5** and **6** previously investigated by us. [1,2-4] It is also to expect that such complexes would be biologically active, since the ligands possess prominent such properties. [12]

In this paper I report the preparation and characterization of Cu(II) complexes of positively-charged derivatives of aminoglutethimide of type **8**. The new complexes were characterized by elemental analysis, IR and electronic spectroscopy, as well as magnetic moment measurements, conductimetric and thermogravimetric data. They were also tested for their ability to inhibit CA, showing weak such properties.

### Materials and Methods

IR spectra were obtained KBr pellets, with a Beckmann 4260 instrument, in the range 200 - 4000 $cm^{-1}$. Electronic spectra were obtained by the diffuse reflectance technique in MgO as reference, with a Perkin Elmer Lambda 17 apparatus. Conductimetric measurements were done in DMF solutions, at 25°C (concentrations of 1 mM of complex) with a Fisher conductimeter. Magnetic susceptibility measurements were done at room temperature by Faraday's method, using $CoHg(NCS)_4$ as standard. Elemental analyses were done by combustion for C,H,N with an automated Carlo Erba analyzer, and gravimetrically for the metal ion, and were ± 0.5% of the theoretical values. Thermogravimetric measurements were done in air, at a heating rate of 10°C/min., with a Perkin Elmer 3600 thermobalance.

Aminoglutethimide **7** (pure *L* stereoisomer) used in the syntheses was from Sigma. Pyrylium perchlorates used to prepare derivatives **8** (by reaction with aminoglutethimide) were synthesized by literature procedures, by bisacylation of olefins or their precursors, as originally described by Nenitzescu and Balaban[17], from commercially available raw materials. Compounds **8** were synthesized as reported previously, [12] from **7** and trisubstituted pyrylium salts in alcohol, by the general method involved in reaction of such reagents with N-nucleophiles.[18] Copper(II) perchlorate was from Aldrich and was used without additional purification. Bovine CA II and human CA I were from Sigma Chemical Co. Inhibitors were assayed by Maren's micromethod, in the conditions of the E-I (enzyme-inhibitor) technique, at 0°C in veronal buffer. [19] $IC_{50}$ values represent the molarity of inhibitor producing a 50% decrease of CA specific activity for the $CO_2$ hydration reaction. [5,19]

### Synthesis of coordination compounds 9a-f

An amount of 20 mMoles of pyridinium perchlorate **8a-f** was suspended in 50 mL of ethanol and 20 mMoles of NaOH dissolved in 10 mL water were added. This solution was treated with an aqueous solution obtained by dissolving 10 mMoles of Cu(II) perchlorate in 10 mL of water, under intense stirring. The reaction mixture was stirred at room temperature for 1 hour, complexes **9a-f** which precipitated were filtered and air-dried. Yields were of 75-80%.

### Results and Discussion

Compounds **8a-f** used in this study were previously reported, [12] and were obtained by reaction of 2,4,6-trisubstituted pyrylium salts with aminoglutethimide **7**.[18] Compounds used in the present study, shown in Table I together with some selected IR bands (relevant for their complexation behavior), possessed aliphatic, aromatic and the combination of these two moieties in their molecules, which were chosen in order to investigate the role of substitution pattern upon properties and biological activity of the obtained complexes.

Table I: Compounds **8a-f** used for the preparation of Cu(II) complexes and some of their selected IR bands.

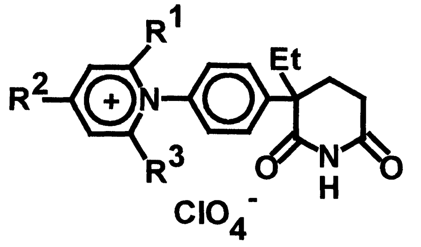

**8**

| 8 | $R^1$ | $R^2$ | $R^3$ | IR Bands [a] ($cm^{-1}$) | | | |
|---|---|---|---|---|---|---|---|
| | | | | amide I[b] | amide II[c] | amide III[d] | ν (NH)[e] |
| **a** | Me | Me | Me | 1690 | 1520 | 1295 | 3060 |
| **b** | *i*-Pr | Me | *i*-Pr | 1695 | 1520 | 1300 | 3060 |
| **c** | *t*-Bu | Me | *t*-Bu | 1690 | 1535 | 1290 | 3070 |
| **d** | Me | Ph | Me | 1690 | 1500 | 1280 | 3050 |
| **e** | Me | Ph | Ph | 1690 | 1500 | 1270 | 3050 |
| **f** | Ph | Ph | Ph | 1700 | 1520 | 1270 | 3050 |

[a] In KBr; [b] Strong and sharp band; [c] Medium intensity band; [d] Weak band; [e] Weak and broad band.

Complexes **9** were prepared from Cu(II) perchlorate and the sodium salts of **8** (obtained *in situ* from **8** and the stoichiometric amount of NaOH), in molar ratios of 1:2. The blue-green complexes obtained in this way are shown in Table II, together with their elemental analysis and thermogravimetric data (±0.5

% of the theoretical values for Cu, C, H, N and water).

Table II: Complexes **9a-f** of the type $[CuL_2(OH_2)_4](ClO_4)_2$ prepared and their elemental analysis data. L stands for the deprotonated species of derivatives **8a-f**. Moieties $R^1$-$R^3$ in compounds **9a-f** are those originally present in the corresponding derivatives **8**.

| 9 | Color | M.p. (°C) | %Cu[a] | Analysis (calc./found) %C[b] | %H[b] | %N[b] | $\%H_2O^c$ |
|---|---|---|---|---|---|---|---|
| a | blue | 287-9[d] | 6.3/6.1 | 50.0/49.8 | 5.5/5.2 | 5.5/5.4 | 7.1/6.9 |
| b | blue | 261-3[d] | 5.9/5.8 | 56.5/56.4 | 6.8/6.7 | 5.3/5.2 | 6.8/6.8 |
| c | blue | 228-9 | 5.8/5.5 | 59.4/59.3 | 7.3/7.3 | 5.1/5.1 | 6.6/6.4 |
| d | smaragd | 244-6[d] | 5.9/5.5 | 58.4/58.1 | 5.6/5.5 | 5.2/5.0 | 6.7/6.3 |
| e | green | 271-4[d] | 5.6/5.4 | 65.8/65.7 | 5.7/5.7 | 4.9/4.8 | 6.3/6.0 |
| f | green | 288-9[d] | 5.3/5.2 | 72.4/72.1 | 5.7/5.5 | 4.7/4.5 | 6.0/6.1 |

[a]By gravimetry; [b]By combustion; [c] By thermogravimetric (TG) analysis; all four water molecules are lost in a single step, between 160-180°C; d With decomposition; compounds 9 were recrystallized from isopropanol-water (4:1, v/v).

Spectroscopic (IR and electronic), magnetic and conductimetric data of complexes **9a-f** are presented in Table III.

In the IR spectra of the prepared complexes **9**, the following modifications were detected, as compared to the spectra of the corresponding derivatives **8**: (i) the absence of ν(NH) vibrations, which for **8a-f** appeared at 3050-3070 $cm^{-1}$; (ii) the shift of the very intense "amide I" band (ν(C=O) ) with 20-30 $cm^{-1}$ towards lower wavenumbers for complexes **9** (Table III) as compared to the corresponding vibrations of derivatives **8** (Table I); (iii) the other amide bands ("amide II" around 1500-1520 $cm^{-1}$, and "amide III" around 1270-1300 $cm^{-1}$ in the ligands) appear in complexes **9** at almost the same wavenumbers (data not shown), but they are not so well resolved and have a reduced intensity; (iv) the very intense perchlorate bands, at 625 and 1100 $cm^{-1}$, are present in both compounds **8** and **9**; (v) at wavenumbers under 400 $cm^{-1}$, all complexes **9** show low intensity bands at 380 and 290 $cm^{-1}$, tentatively assigned as due to Cu-O and Cu-N stretching vibrations.[20,21]

Table III: Spectroscopic, magnetic and conductimetric data of complexes **9a-f**.

| **9** | **IR. Spectra** [a], $cm^{-1}$ ν(M-L) | ν(C=O) | **Electronic Spectra** [b], $\lambda_{max}$, ($cm^{-1}$) | $\mu_{eff}$[c] (BM) | **Conductimetry**[d] ($\Omega^{-1}.cm^2 \cdot M^{-1}$) |
|---|---|---|---|---|---|
| **a** | 290; 380 | 1670 | 14,300 | 2.29 | 287 |
| **b** | 290; 380 | 1670 | 14,320 | 2.26 | 293 |
| **c** | 290; 380 | 1660 | 14,200 | 2.30 | 298 |
| **d** | 290; 380 | 1660 | 14,400 | 2.20 | 275 |
| **e** | 290; 380 | 1670 | 14,200 | 2.24 | 280 |
| **f** | 290; 380 | 1670 | 14,200 | 2.25 | 265 |

[a] In KBr; [b] By diffuse reflectance in MgO as standard; [c] At room temperature; d In DMF, at 25°C, $10^{-3}$ M solution.

The magnetic moment data (at room temperature) of the new complexes - around 2.2-2.3 BM - are typical for Cu(II) in octahedral surrounding.[20,21] In the diffuse reflectance spectra of these complexes a very broad and large band, centered at 14,200-14,400 $cm^{-1}$ was detected, which is probably due to intense charge-transfer effects occurring with this type of positively-charged ligands, [3,7] so that the specific d-d transitions of Cu(II) are not seen. On the other hand, conductimetric measurements in DMF solutions of complexes **9** show a 1:2 electrolyte type of behavior for all of them, with molar conductibilities around 300 $\Omega^{-1}.cm^2.M^{-1}$.[3,10]

From the above data it can be concluded that ligands **8** act monodentately, through the ionized imidic nitrogen, similarly with saccharin **5**[3,4,22] and diazoxide **6**,[1] for which similar complexes were reported. Taking into account the presence of four coordinated water molecules in complexes **9**, the structure proposed for the new complexes is shown bellow and is similar to the structure of the saccharin complex $[Cu(sac)_2(OH_2)_4]$ which was determined by means of X-ray crystallography.[22]

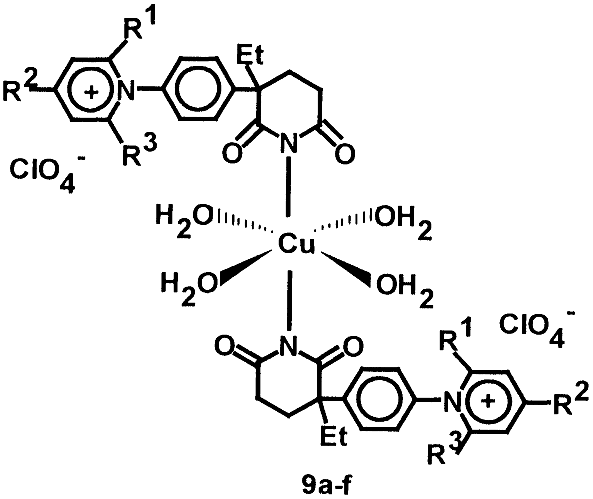

**9a-f**

The prepared complexes as well as the original ligands were tested for their ability to inhibit CA isozymes I and II by Maren's micromethod.[19] Inhibition data with compounds **8**,**9** and standard inhibitors for comparison are presented in Table IV.

Table IV : CA I and II inhibition data, for $CO_2$ hydration, with compounds **1**, **5-17**, determined by Maren's method.[19] For comparison data of a strong (**1**) and a weak (**5**) CA inhibitor are also included.

| Compound | $IC_{50}$ (μM)[a] | |
|---|---|---|
| | CA I | CA II |
| **1** | 0.2[b] | 0.07[b] |
| **5** | 188 | 97[c] |
| **8a** | 450 | 380 |
| **8b** | 420 | 310 |
| **8c** | 445 | 340 |
| **8d** | 390 | 215 |
| **8e** | 850 | 540 |
| **8f** | 970 | 580 |
| **9a** | 105 | 68 |
| **9b** | 95 | 82 |
| **9c** | 90 | 60 |
| **9d** | 105 | 45 |
| **9e** | 440 | 360 |
| **9f** | 485 | 390 |

[a] Molarity of inhibitor producing a 50% decrease of enzyme specific activity for the $CO_2$ hydration reaction, at 0°C;[b] From refs.[5]; [c] From ref.[8a]

From data of Table IV it can be seen that, as expected, derivatives **8**,**9** behave as weak inhibitors of both CA isozymes. Still, it is obvious that the substitution pattern of the pyridinium ring strongly influences biological activity, as for positively-charged sulfonamides of type **4** possessing the same type of groups in their molecules.[7] Thus, derivatives **8** are extremely weak inhibitors for both isozymes, the strongest one being the 2,6-dimethyl-4-phenyl-pyridinium substituted compound **8d** (significant inhibition in the millimolar range). The Cu(II) complexes **9** behave as much stronger inhibitors, presumably due to a dual inhibition, as observed for the metal complexes of heterocyclic sulfonamides.[1-4] As expected, complexes **9** inhibit these two isozymes 2 to 6 times stronger as compared to the corresponding compounds **8** from which they are derived. The strongest inhibitor was always the one containing the 4-phenyl-2,6-dimethyl moiety, followed by the complexes possessing only aliphatic groups at the pyridinium ring. In contrast, the weakest inhibitors were those possessing more than one aromatic group in that position (**8e,f** and **9e,f**, respectively). This is probably due to the very bulky nature of the last derivatives and an impaired access within the CA active site.

### References

1. Part 1 of this series: C.T.Supuran, *Metal Based Drugs, in press.*
2. a) C.T.Supuran, in "Carbonic Anhydrase and Modulation of Physiologic and Pathologic Processes in the Organism", I.Puscas Ed., Helicon, Timisoara 1994, pp. 29-111; b) G.Alzuet, S.Ferrer, J.Borras and C.T.Supuran, *Roum.Chem.Quart.Rev.*, **1994**, *2*, 283-300.
3. a) C.T.Supuran, M.Andruh, and I.Puscas, *Rev.Roum.Chim.*, **1990**, *35*, 393-398; b) S.Ferrer, A.Jimenez and J.Borras, *Inorg.Chim.Acta*, **1987**, *129*, 103-106; c) S.Ferrer, G.Alzuet and J.Borras, *J.Inorg.Biochem.*, **1989**, *37*, 163-174; d) C.T.Supuran, G.Manole and I.Manzatu, *Rev.Roum.Chim.*, **1992**, *37*, 739-744; e) C.T.Supuran, G.Manole and M.Andruh, *J.Inorg.Biochem.*, **1993**, *49*, 97-104; f) C.T.Supuran and M.Andruh, *Rev.Roum.Chim.*, **1994**, *39*, 1229-1234.
4. C.T.Supuran,.*Roum.Chem.Quart.Rev.*, **1993**, *1*, 77-116.
5. a)T.H.Maren, *Physiol.Rev.*, **1967**, *47*, 595-781; b) T.H.Maren, *Drug Dev.Res.*, **1987**, *10*, 255-276.
6. C.T.Supuran and I.Puscas, in "Carbonic Anhydrase and Modulation of Physiologic and Pathologic Processes, in the Organism", I.Puscas Ed., Helicon, Timisoara 1994, pp. 113-146.
7. a) C.T.Supuran, G.Manole, A.Dinculescu, A.Schiketanz, M.D.Gheorghiu, I.Puscas, and A.T.Blaban, *J.Pharm.Sci.*, **1992**, *81*, 716-719; b) C.T.Supuran and B.W.Clare, *Eur.J.Med.Chem.*, **1995**, *30*, 687-696.
8. a) C.T.Supuran and M.D.Banciu, *Rev.Roum.Chim.*, **1991**, *36*, 1345-1353; b) C.T.Supuran, G.Loloiu and G.Manole, *Rev.Roum.Chim.*, **1992**, *37,* 1181-1189.
9. D.L.Pain, B.J.Peart and K.R.H.Woolridge, in "Comprehensive Heterocyclic Chemistry", A.R.Katritzky and C.W.Rees Eds., Pergamon Press, New York, 1984, Vol 6 Part 4B, pp 131-175.
10. L.Antolini, L.P.Battaglia, G.Battistuzzi Gavioli, A. Bonamartini Corradi, G.Grandi, G.Marcotrigiano, L.Menabue and G.C.Pellacani, *J.Am.Chem.Soc.*, **1983**, *105*, 4327-4332.
11. T.Kowalik-Jankowska, H.Kozlowski, L.D.Pettit, K.Pawelczak and M.Makowski, *J.Inorg.Biochem.*, **1995**, *57*, 183-190.
12. C.T.Supuran, D.Stoicescu, O.Maior and A.T.Balaban, *Rev.Roum.Chim.*, **1993**, *38*, 605-612.
13. P.Calabresi and B.A.Chabner, in "The Pharmacological Basis of Therapeutics", 8th Edition, A.G.Gilman, T.W.Rall, A.S.Nies and P.Taylor Eds., Pergamon, New York, 1990, pp. 1209-1263.
14. R.J.Santen, E.Samolijk and S.A.Wells, *J.Clin.Endocrinol.Metabol.*, **1980**, *51*, 473-477.
15. E.M.Gold, *Ann.Intern.Med.*, **1979**, *90*, 829-844.
16. R.C.Haynes Jr., in "The Pharmacological Basis of Therapeutics", 8th Edition, A.G.Gilman, T.W.Rall, A.S.Nies and P.Taylor Eds., Pergamon, New York, 1990, pp. 1431-1462.
17. a) A.T.Balaban and C.D.Nenitzescu, *Liebigs Ann.Chem.*, **1959**, *625*, 74-88; b). C.T.Supuran, I.Baciu and A.T.Balaban, *Rev.Roum.Chim.*,**1993**, *38*, 725-732.
18. A.T.Balaban, A.Dinculescu, G.N.Dorofeenko, G.W.Fischer, A.V.Koblik, V.V.Mezheritskii and W.Schroth, "Pyrylium Salts: Syntheses, Reactions and Physical Properties", Academic, New York, 1982.
19. T.H.Maren, *J.Pharmacol.Exp.Ther.*, **1960**, *130*, 26-30.
20. D.A.Thornton, *Coord.Chem.Rev.*, **1990**, *104*, 251-259.
21. E.S.Raper, *Coord.Chem.Rev.*, **1994**, *129*, 91-156.
22. K.J.Ahmed, A.Habib, S.C.Haider, K.M.A.Malik and M.B.Hursthouse, *Inorg.Chim.Acta*, **1981**, *56*, L-37.

**Received: November 24, 1995 - Accepted: December 22, 1995 - Received in revised camera-ready format: January 15, 1996**

